# Supplementary material for: Effect of internal surface structure of the north wall on Chinese solar greenhouse thermal microclimate based on computational fluid dynamics
Source: PLoS One. 2020 Apr 15;15(4):e0231316. doi: 10.1371/journal.pone.0231316 (PMC7159206; doi:10.1371/journal.pone.0231316)
Supplement: S1 Table — (DOCX) [file pone.0231316.s001.docx]

| **Nomenclature** | | | |
| --- | --- | --- | --- |
| *a* | absorption coefficient | $\vec{v}$ | velocity vector |
| *C_i_* | constant | *Y_M_* | contribution of the fluctuating dilatation in compressible turbulence to the overall dissipation rate |
| $c$ | specific thermal capacity (J m^-3^ °C^-1^) | $\alpha$ | heat transfer coefficient (W m^-2^ °C^-1^) |
| *D* | thermal inertia index | $\beta$ | thermal expansion coefficient |
| *E* | flow energy (N m) | $\varepsilon$ | turbulent dissipation rate (m^2^ s^-3^) |
| *G_k_* | generation of turbulence kinetic energy due to the mean velocity gradients (N m) | $\lambda$ | thermal conductivity (W m^-1^ °C^-1^) |
| *G_b_* | generation of turbulence kinetic energy due to buoyancy (N m) | $\mu_{t}$ | turbulent viscosity |
| $\vec{g}$ | gravitational acceleration (m s^-2^) | $\rho$ | density of air (kg m^-3^) |
| *H* | heat flux (W m^-2^) | $\sigma_{s}$ | scattering coefficient |
| *h_j_* | species enthalpy | $\sigma_{k}$ | Turbulent Prandtl numbers for k |
| *I* | incident radiation | $\sigma_{\varepsilon}$ | Turbulent Prandtl numbers for ε |
| $\vec{J}_{j}$ | diffusion flux of species (kg m^-2^ s) | $\bar{\bar{\tau}}_{eff}$ | effective stress tensor (Pa) |
| *k* | turbulence kinetic energy (m^2^ s^-2^) | $\Phi$ | diffusion phase function |
| *k_eff_* | effective conductivity (W m^-1^ °C) | **Abbreviations** | |
| *n* | refractive index of the medium | $\Omega^{'}$ | solid angle |
| *P* | static pressure (Pa) | AW | alveolate wall |
| *q* | heat transfer (J m^-2^) | CCW | concave-convex wall |
| $\vec{r}$ | solar position vector | CFD | computational fluid dynamics |
| *S* | thermal storage coefficient (W m^-2^ °C ^-1^) | CSG | Chinese solar greenhouses |
| *S_i_* | user-defined source term | HW | horizontal wall |
| $\vec{s}$ | solar direction vector | PW | plane wall |
| *t* | time (s) | VW | vertical wall |
